# Soybean Cyst Nematode Resistance Emerged via Artificial Selection of Duplicated Serine Hydroxymethyltransferase Genes

**DOI:** 10.3389/fpls.2016.00998

**Published:** 2016-07-08

**Authors:** Xiao-Yi Wu, Guang-Can Zhou, Yun-Xia Chen, Ping Wu, Li-Wei Liu, Fang-Fang Ma, Mian Wu, Cheng-Chen Liu, Ying-Jie Zeng, Alexander E. Chu, Yue-Yu Hang, Jian-Qun Chen, Bin Wang

**Affiliations:** ^1^Laboratory of Plant Genetics and Molecular Evolution, School of Life Sciences, Nanjing University, NanjingChina; ^2^Institute of Botany, Jiangsu Province and Chinese Academy of Sciences, NanjingChina; ^3^Department of Plant and Wildlife Sciences, Brigham Young University, Provo, UTUSA

**Keywords:** *Rhg4*, *SHMT*, gene duplication, artificial selection, evolution

## Abstract

A major soybean (*Forrest* cultivar) quantitative trait locus (QTL) gene, *Rhg4*, which controls resistance to soybean cyst nematodes (SCN), encodes the enzyme serine hydroxylmethyltransferase (SHMT). The resistant allele possesses two critical missense mutations (P130R and N358Y) compared to that of the sensitive allele, *rhg4*. To understand the evolutionary history of this gene, sequences of 117 *SHMT* family members from 18 representative plant species were used to reconstruct their phylogeny. According to this phylogeny, the plant SHMT gene family can be divided into two groups and four subgroups (Ia, Ib, IIa, and IIb). Belonging to the Subgroup Ia lineage, the *rhg4* gene evolved from a recent duplication event in *Glycine* sp.. To further explore how the SCN-resistant allele emerged, both the *rhg4* gene and its closest homolog, the *rhg4h* gene, were isolated from 33 cultivated and 68 wild soybean varieties. The results suggested that after gene duplication, the soybean *rhg4* gene accumulated a higher number of non-synonymous mutations than *rhg4h*. Although a higher number of segregating sites and gene haplotypes were detected in wild soybeans than in cultivars, the SCN-resistant *Rhg4* allele (represented by haplotype 4) was not found in wild varieties. Instead, a very similar allele, haplotype 3, was observed in wild soybeans at a frequency of 7.4%, although it lacked the two critical non-synonymous substitutions. Taken together, these findings support that the SCN-resistant *Rhg4* allele likely emerged via artificial selection during the soybean domestication process, based on a SCN-sensitive allele inherited from wild soybeans.

## Introduction

Essential for cellular one-carbon metabolism, serine hydroxymethyltransferase (SHMT, EC 2.1.2.1) is a ubiquitous enzyme present in all organisms. It catalyzes the reversible conversions of serine and glycine, thereby providing one-carbon units for a series of important biosynthetic processes such as the syntheses of methionine, thymidylates, and purines (Schirch, 1982; Matthews and Drummond, 1990; Bauwe and Kolukisaoglu, 2003; Schirch and Szebenyi, 2005). In bacteria and Archaea, a single gene encodes the SHMT enzyme, which exhibits a homodimeric structure (Scarsdale et al., 2000; Trivedi et al., 2002; Angelucci et al., 2014), whereas in animals and fungi, two genes encode two SHMT isoforms (cytosolic and mitochondrial), which are organized as tetramers (Martini et al., 1987, 1989; Garrow et al., 1993; McNeil et al., 1994; Kastanos et al., 1997).

A higher number of *SHMT* genes have been reported in green plants (McClung et al., 2000; Bauwe and Kolukisaoglu, 2003; Zhang et al., 2010). Up to seven *SHMT* genes (*AtSHM1*–*AtSHM7*) were identified in *Arabidopsis thaliana.* These genes are predicted to encode various SHMT isoforms that function in different cellular compartments, with AtSHM1 and AtSHM2 enzymes working in the mitochondria, AtSHM3 targeting the chloroplast, AtSHM4 and AtSHM5 playing roles in the cytosol, and AtSHM6 and AtSHM7 acting in the nuclei (McClung et al., 2000; Bauwe and Kolukisaoglu, 2003). Studies on these *Arabidopsis* SHMT proteins, on one hand, have confirmed their cellular locations (for AtSHM1–AtSHM4 isoforms); on the other hand, these investigations have also revealed interesting roles of SHMTs in influencing plant defense abilities (Moreno et al., 2005; Voll et al., 2006; Zhang et al., 2010; Engel et al., 2011; Wei et al., 2013). One example is the *Arabidopsis SHM1* gene. Mutants of this gene have been determined to be more susceptible than wild-type plants to a number of pathogen infections (Moreno et al., 2005). In addition, another *SHMT* gene named *Rhg4* was recently characterized from the soybean cultivar Forrest and was determined to confer resistance to soybean cyst nematode (*Heterodera glycines*, SCN) (Liu et al., 2012). Compared to sensitive alleles, this resistant allele contains two critical point mutations resulting in two amino acid changes (P130R and N358Y), which were hypothesized to impair a key regulatory property of the encoded SHMT enzyme. The altered enzyme may further influence the folate homeostasis in soybean root cells, and ultimately restrict the growth of cyst nematodes (Liu et al., 2012). This resistant *Rhg4* allele was also detected in a few other soybean cultivars including Peking, and overexpression of *Rhg4-Peking* in roots of SCN-sensitive cultivar Williams82 greatly reduced the nematode infections (Matthews et al., 2013). Therefore, in either *Arabidopsis* or soybean, SHMT seems to be involved in host defense. But the relationship between the characterized soybean *SHMT* gene (*Rhg4*) and the *Arabidopsis AtSHM1* gene is still undetermined. Also, the frequency of the *Rhg4* allele in wild soybean populations (*Glycine soja*) has not been investigated, and the question of how such SCN-resistance evolved remained elusive.

To understand how and why many plants evolved multiple *SHMT* genes and to clarify their relationships, the present study initially surveyed 18 representative plant genomes to identify all *SHMT* genes, and then reconstructed a robust phylogenetic tree using the maximum likelihood (ML) method. Moreover, to explore how the SCN-resistant allele of the *SHMT* gene evolved, up to 33 soybean cultivars (*G. max*) and 68 wild soybeans (*G. soja*) were used to isolate the *Rhg4* gene as well as its close homolog.

## Materials and Methods

### SHMT Sequence Collections from Representative Plant Genomes

Using the *Arabidopsis* SHMT protein sequences (AtSHM1–AtSHM7) as query sequences and performing BLASTP and TBLASTN searches, the *SHMT* genes in 17 other representative plant genomes were identified and downloaded from the Phytozome database (version 10.3)^1^ as well as the spruce genome database^2^. The 17 representative plant genomes included *Chlamydomonas reinhardtii* (v5.5), *Ostreococcus lucimarinus* (v2.0), *Physcomitrella patens* (v3.1), *Selaginella moellendorffii* (v1.0), *Picea abies* (v1.0), *Amborella trichopoda* (v1.0), *Oryza sativa* (v7.0), *Brachypodium distachyon* (v2.1), *Sorghum bicolor* (v2.1), *A. lyrata* (v1.0), *Brassica rapa* (v1.3), *Medicago truncatula* (Mt4.0v1), *G. max* (Wm82.a2.v1), *Phaseolus vulgaris* (v1.0), *Mimulus guttatus* (v2.0), *Solanum lycopersicum* (iTAGv2.3), and *S. tuberosum* (v3.4) (Supplementary Table S1).

### Sequence Alignment and Phylogenetic Analysis

The predicted exon-intron structures of the identified *SHMT* genes were manually examined to identify annotation errors. Then, the coding sequences were aligned by using ClustalW, as implemented in Mega 5.0 (Tamura et al., 2011). To improve sequence alignment, the sequences were aligned under the guide of their amino acid sequences and the *SHMT* gene of *Escherichia coli* O157:H7 strain Sakai (NC_002695) was used as outgroup. Based on the obtained alignment, a *SHMT* phylogenetic tree was reconstructed by using the ML method, as provided in Mega 5.0 (Tamura et al., 2011). A total of 100 bootstrap replicates were performed to test the robustness of the internal branches. The tree of the *SHMT* gene family was then carefully examined and compared to a previously reported neighbor-joining tree, which encompassed nine plant species (Zhang et al., 2010).

The positions of the *AtSHM1* and the soybean *rhg4* (*Glyma08g108900* in Williams82, recessive to *Rhg4* in Forrest) on the reconstructed phylogenetic tree were examined to determine their relationships. In addition, to clarify the evolutionary path of the soybean *rhg4* gene, its closely related homologs in leguminous species were assessed and compared.

### Investigating the Evolution of the Soybean *Rhg4* Gene and Its Closely Related Homolog

The soybean *rhg4* gene was classified under subgroup Ia of the *SHMT* gene family and has a closely related homolog, *Glyma05G152100* (designated as *rhg4h* hereafter) in the soybean genome. To investigate how these two duplicated genes evolved in soybean, the following gene-specific primers were designed: *rhg4-F* (5′-CGGCGTTAAACAAATACTAGA-3′, which was located 340 bp upstream of the start codon) and *rhg4-R* (5′-GCCTTAACTTGTTTTGGATCT-3′, situated 1,272 bp downstream of the stop codon); *rhg4h-F* (5′-TCTGACATGTTGAATAGGGTA-3′, located 195 bp upstream of the start codon) and *rhg4h-R* (5′-GATGTGTGTTTTAGGACGCTG-3′, which was positioned 1,045 bp downstream of the stop codon). Next, a number of cultivated soybeans (33 in total) and wild soybean materials (68 in total) were used to isolate the *rhg4* and *rhg4h* genes. These soybean materials were mainly obtained from the National Center for Soybean Improvement of China at the Nanjing Agriculture University, and their details are presented in Supplementary Table S2. The amplified PCR products of the *rhg4* gene (~3.8 kb) and the *rhg4h* gene (~3.5 kb) were purified with a PCR Purification kit (Qiagen, Hilden, Germany) following the manufacturer’s instructions, sequenced bi-directionally (Genescript Co., Nanjing, China), and the contigs assembled and edited using the Sequencher 4.5 software (Gene Codes, Ann Arbor, MI, USA).

The obtained clean-read sequences of both genes were aligned as earlier described. Gene haplotypes of the cultivated soybeans and wild soybeans were compared using the software, DnaSP v5.10.02 (Librado and Rozas, 2009). For polymorphic sites detected within coding regions, the nucleotide mutations were examined to see whether these were synonymous or non-synonymous, and sequence variations in the *rhg4* and *rhg4h* genes were also compared to determine whether the two duplicated genes exhibited different evolutionary patterns.

## Results

### Plant *SHMT* Gene Family Can Be Divided into Two Groups and Four Subgroups

By performing BLASTP and TBLASTN searches, a total of 117 *SHMT* genes were identified in the 18 representative plant genomes (Supplementary Table S1). The green algae, represented by *C. reinhardtii* and *O. lucimarinus*, comprised three *SHMT* genes, whereas land plants consisted of at least four copies, with the highest number, 14 copies, detected in the soybean genome.

**FIGURE 1:**
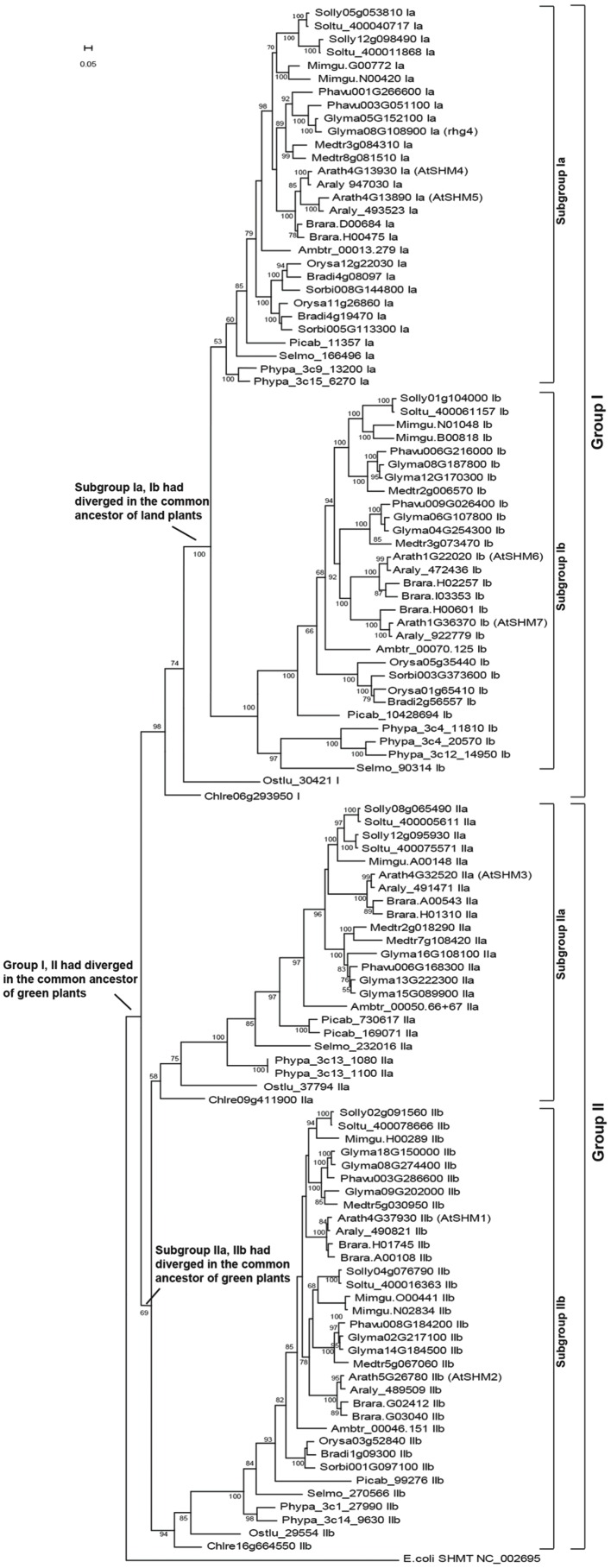
**The reconstructed *SHMT* gene phylogeny of green plants divided into two groups and four subgroups.** A total of 117 *SHMT* genes (Supplementary Table S1) of 18 representative plants were identified, with the *Escherichia coli SHMT* sequence (NC_002695) used as outgroup. The positions of the seven *SHMT* genes in the *Arabidopsis thaliana* genome (*AtSHM1*-*AtSHM7*) and the soybean *rhg4* gene are marked. The bar represents a mutation rate of 0.05 substitutions per site.

Phylogenetic reconstruction of these *SHMT* genes revealed two clear clades, groups I and II, which had separated since the common ancestor of green plants (**Figure 1**). In fact, before the green algae and land plants diverged, the group II lineage also separated into two sublineages (subgroups IIa and IIb, **Figure 1**). For the group I lineage, the present study revealed that the green algae genes, *Chlre06g293950* and *Ostlu_30421* (Supplementary Table S1), were located proximal to the base of the group I clade (**Figure 1**), thereby supporting a hypothesis that only one gene belonging to the group I clade had existed in the common ancestor of green plants. **Figure 1** further shows that in the last common ancestor of land plants, the group I lineage further diverged into two sublineages, subgroups Ia and Ib. Since then, most land plants maintained the four *SHMT* sublineages successfully (Supplementary Table S1; **Figure 1**). For example, in the early vascular plant *S. moellendorffii* and the early flowering plant *A. trichopoda*, four copies of *SHMT* genes were detected, with each belonging to the subgroups Ia, Ib, IIa, and IIb, respectively. For the gymnosperm species *P. abies*, five copies were present, the consequence of a duplication event in subgroup IIa (Supplementary Table S1; **Figure 1**). In monocots, a special gene loss event occurred in sublineage IIa, and a gene duplication event on sublineage Ia had compensated for such a loss. The dicot species showed more plasticity, as each of the four sublineages of *SHMT* genes had gone through duplications. An extreme example is the soybean genome, which retained two copies of subgroup Ia, four copies of Ib, three copies of IIa, and five copies of IIb genes (Supplementary Table S1, **Figure 1**).

### The Soybean SCN-Resistant *SHMT* gene, *Rhg4*, Belongs to the Subgroup Ia

**FIGURE 2:**
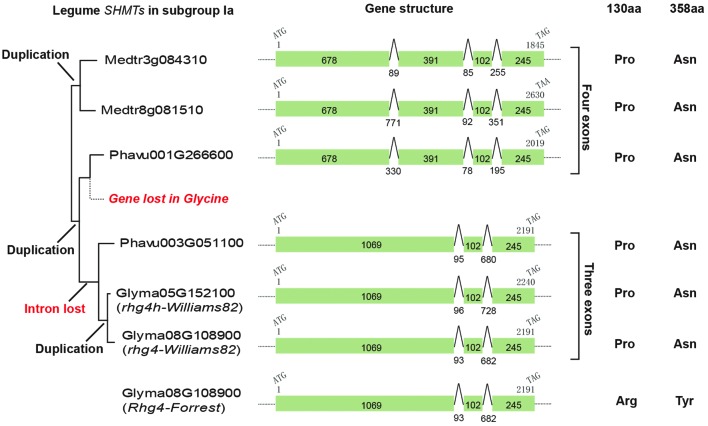
**The evolution of legume subgroup Ia *SHMT* genes.** Left, a partial tree cut from **Figure 1** showing that three gene duplication events and also one gene loss event had occurred. Middle, gene structural analysis showed that an intron loss event had occurred in the common ancestor of *Phavu003G051100* and two soybean genes, *Glyma08G108900* (*rhg4*) and *Glyma05G152100* (*rhg4h*). Right, the amino acids at positions 130 and 389 in each gene are shown.

The *AtSHM1* gene, as well as its closely related homolog, *AtSHM2* (*Arath5G26780*), belongs to the subgroup IIb in the reconstructed SHMT gene phylogeny (**Figure 1**). The soybean *rhg4* gene, however, belongs to the subgroup Ia (**Figure 1**), indicating a distant relationship to *AtSHM1*. The *rhg4* also has a closely related homolog, *Glyma05G152100* (*rhg4h*, Supplementary Table S1). These two soybean genes, together with a common bean gene, *Phavu003G051100*, formed a monophyletic group (**Figures 1** and **2**), suggesting that the two soybean genes were only recently duplicated in the *Glycine* lineage. Interestingly, for all the nine dicot species investigated in the present study, two copies of subgroup Ia *SHMT* genes were retained, and these were all derived from recent, independent duplication events. For example, the two *Arabidopsis* genes of this subgroup, *AtSHM4* (*Arath4G13930*) and *AtSHM5* (*Arath4G13890*), were duplicated in the common ancestor of *A. thaliana* and *A. lyrata* (**Figure 1**), and AtSHM4 has been confirmed to function in the cytosol (Wei et al., 2013). In legumes, a duplication event occurred in the *Medicago* lineage, producing two subgroup Ia genes (*Medtr3g084310* and *Medtr8g081510*), whereas another duplication event occurred in the common ancestor of the *Phaseolus* and *Glycine* lineages (**Figure 2**). While the *Phaseolus* lineage maintained both copies of *SHMT* genes (*Phavu001g266600* and *Phavu003g051100*) that resulted from such a duplication event, the *Glycine* lineage apparently lost one and further duplicated the other one (**Figure 2**). Such evolutionary scenario is also supported by examining their gene organizations. The two *Medicago* subgroup Ia *SHMT* genes and the common bean gene *Phavu001g266600* exhibited a four-exon structure (**Figure 2**), which represents the ancenstral structural organization of subgroup Ia *SHMT* genes and was conserved among other non-legume angiosperms, including *A. trichopoda* and monocots (data not shown). The common bean gene *Phavu003g051100* and the duplicated soybean genes *rhg4* and *rhg4h*, however, had lost the first intron, thereby exhibiting a derived three-exon structure (**Figure 2**).

We further examined the amino acid sequences of these SHMTs, particularly the two amino acid positions (130 and 358) that are critical for SCN resistance in certain soybean cultivars (Liu et al., 2012). Both *Medicago* and *Phaseolus* subgroup Ia SHMTs possessed a Pro residue at position 130 and an Asn at position 358, which is same as those of the SCN-sensitive allele of the soybean gene *rhg4* and its homolog, *rhg4h* (**Figure 2**). Actually, the Pro residue at position 130 is conserved among all the four subgroups of SHMTs, whereas the Asn residue at position 358 is conserved among all subgroup Ia sequences (Supplementary Figure S1).

### The Evolution of *rhg4* and *rhg4h* Genes in Soybeans Showed Evidence of Varied Functional Constraints

In the soybean genome, the *rhg4* gene and its homolog *rhg4h* are on chromosomes 8 and 5, respectively (Supplementary Table S1, **Figure 2**). Examination of their neighboring genes revealed a good syntenic relationship, suggesting that *rhg4* and *rhg4h* were derived from a whole genome duplication event that occurred in the *Glycine* lineage approximately 13 million years ago (MYA; Schmutz et al., 2010). Possessing the same gene structures and the same length (1,416 bp) of coding sequences (**Figure 2**), the two genes exhibited a total of 56 nucleotide differences, among which only six were non-synonymous. The calculated non-synonymous rate (dn) and synonymous rate (ds) was 0.007 and 0.126, respectively, with a dn/ds ratio of 0.056.

**FIGURE 3:**
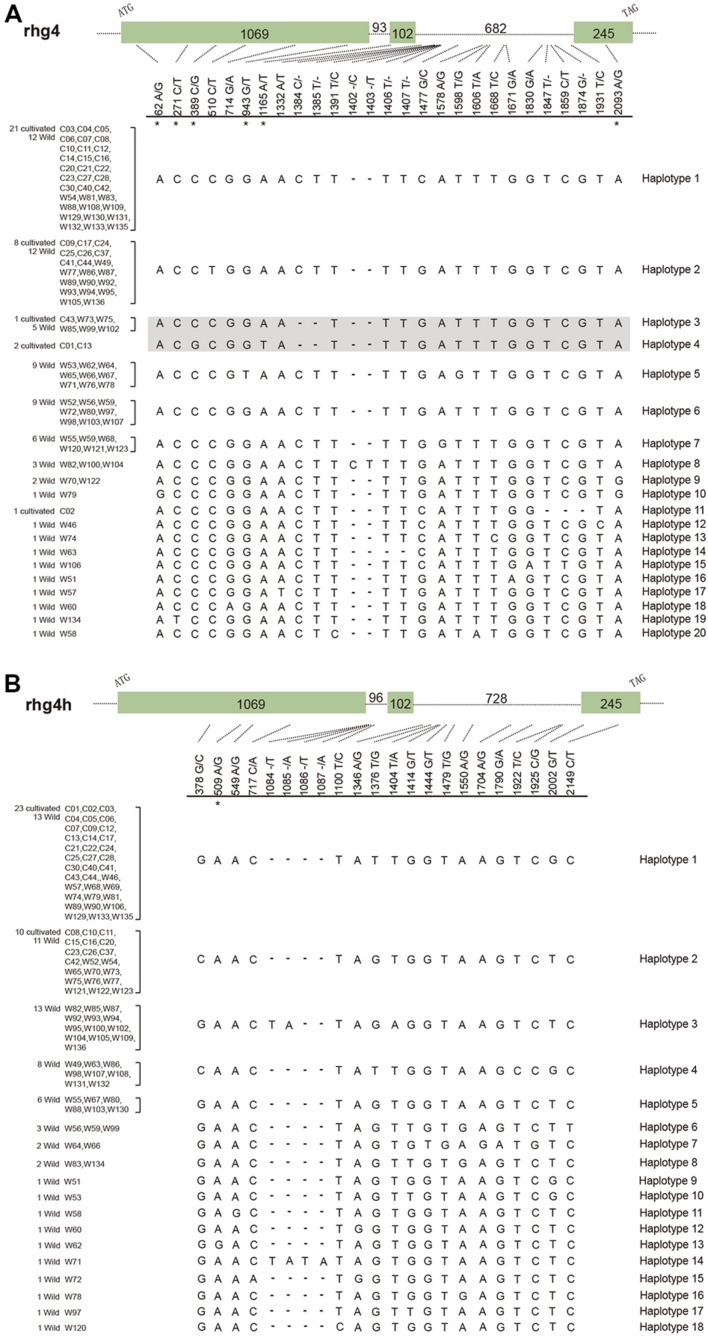
**Gene haplotypes detected in soybean populations. (A)** *Rhg4* gene, with 27 segregating sites and 20 haplotypes detected. Haplotype 4 representing the SCN-resistant allele (*Rhg4*) and its closely related allele (haplotype 3) are shown in shadow. **(B)** *Rhg4h* gene, with 22 segregating sites and 18 haplotypes detected. Six non-synonymous substitution sites in the *rhg4* gene and one in the *rhg4h* gene are labeled with asterisks.

To further explore the evolutionary patterns of the *rhg4* and *rhg4h* genes in soybean populations, we designed gene-specific primers and successfully amplified both genes from a total of 33 cultivated and 68 wild soybeans (Supplementary Table S2). Analysis of the obtained clean-read sequences (101 each) identified 27 segregating sites in the *rhg4* gene and 22 in the *rhg4h* gene (**Figure 3**). For both genes, a higher level of polymorphism was observed in the wild soybeans than in the cultivars. Among the 20 haplotypes detected in the *rhg4* gene, only five (haplotypes 1, 2, 3, 4, and 11) were observed in the cultivars. The *rhg4h* gene showed more bias, with only two haplotypes (1 and 2) detected in cultivated soybeans and a total of 18 in wild varieties (**Figure 3**).

We also explored whether the *rhg4* and *rhg4h* genes in soybean populations experienced a similar degree of functional constraint after duplication. Eight nucleotide substitutions were detected in the coding regions of the *rhg4* gene among soybean population, with seven that occurred in exon 1 and one in exon 3 (**Figure 3**). Interestingly, six of these substitutions resulted in amino acid changes, with a ratio (6 non-synonymous vs. 2 synonymous) that was apparently higher than that observed between the *rhg4* and *rhg4h* genes (6 non-synonymous vs. 50 synonymous), and highly significant *G*-test results (*G* = 15.20, *P* = 0.000048). On the other hand, for the *rhg4h* genes, six segregating sites were detected in coding regions, of which five were synonymous, and the ratio (1 non-synonymous vs. 5 synonymous) did not significantly deviate from that between *rhg4* and *rhg4h* genes (*G* = 0.038, *P* = 0.85). Therefore, the two duplicated subgroup Ia *SHMT* genes seem underwent different degrees of functional constraint, with the *rhg4* gene showing a higher rate of non-synonymous substitutions in soybean populations.

### The Soybean SCN-Resistant *Rhg4* Allele Is Not Found in Wild Soybeans

In the present study, the detection of a total of 20 haplotypes of *rhg4* genes in soybean populations revealed that the SCN-resistant allele, *Rhg4*, originally characterized in the cultivar *Forrest* (Liu et al., 2012), belonged to the haplotype 4 (**Figure 3**). Although a generally higher level of sequence diversity was observed in wild soybeans compared to cultivars, wild varieties investigated by this study do not harbor haplotype 4. However, haplotype 3 is identical to haplotype 4 except for the two critical amino acid changes at positions 130 and 358 (**Figure 3**). Five wild soybean materials sampled from Sichuan (W73), Yunnan (W75), and three sites in Heilongjiang (W85, W99, and W102) possessed the haplotype 3 allele, suggesting that this allele is present in wild soybeans at a frequency of 7.4%, which is not rare. In addition, cultivar PI88788 (C43) possessed the haplotype 3 too, indicating that this allele had been introduced into certain cultivars. Some other haplotypes (5, 9, and 10) possessing additional amino acid changes were also revealed by our data, especially the haplotype 5, which was detected in 9 wild soybeans collected from Southern China regions (**Figure 3**, Supplementary Table S2). The nucleotide substitution (G to T on site 943) resulted in the Ala residue at position 315 changed to Ser in this haplotype (**Figure 3**). With an occurring frequency of 13.2% in wild soybeans, the potential function of this *SHMT* allele is intriguing to explore.

## Discussion

During pathogen invasions, the plant primary metabolism processes were often modified and their defense abilities could be reinforced (as reviewed by Bolton, 2009; Rojas et al., 2014). As a critical enzyme for providing one-carbon units for many important cellular activities (Schirch, 1982; Matthews and Drummond, 1990; Bauwe and Kolukisaoglu, 2003; Schirch and Szebenyi, 2005), SHMT seems to be one of such primary metabolism enzymes, as recent studies on the *Arabidopsis AtSHM1* gene (*Arath4G37930*) and soybean *Rhg4* gene reported their interesting roles in plant defenses (Moreno et al., 2005; Liu et al., 2012).

To explore the relationship of *Arabidopsis SHM1* and the soybean *Rhg4* genes, in this study, we initially surveyed 117 members from 18 representative plant species and reconstructed a robust *SHMT* gene phylogeny (Supplementary Table S1, **Figure 1**). It was clearly showed that the *Arabidopsis SHM1* gene belongs to the subgroup IIb while the soybean *rhg4* gene belongs to the subgroup Ia, suggesting that the two genes are distantly related, and their roles in defense are unlikely the same. Repeated *SHMT* gene duplication events were also observed at various plant evolutionary nodes (**Figure 1**), making one wonder what the driving forces underlying such patterns were. The early appearance of four sublineages of *SHMT* genes in land plants may reflect an advantage of using different SHMT isoforms to provide one-carbon units in various cellular compartments such as the cytosol (subgroup Ia), nuclei (subgroup Ib), chloroplast (subgroup IIa), and mitochondria (subgroup IIb), as indicated by the studies performed on *A. thaliana* SHMTs (Moreno et al., 2005; Voll et al., 2006; Zhang et al., 2010; Engel et al., 2011; Wei et al., 2013). However, for the sublineage-specific duplications, the functional implications were unclear. Both being functional enzymes working in the mitochondria (subgroup IIa), AtSHM1 and AtSHM2 are closely related homologs and are revealed to operate in a redundant manner in non-photorespiring vascular tissues (Engel et al., 2011), while only AtSHM1 plays photorespiration function in the mesophyll cells of leaves (Voll et al., 2006). In the *AtSHM1* mutants, some defense genes induced by salicylic acid and genes involved in H_2_O_2_ detoxification were expressed, reflecting a response to the excessive production of reactive oxygen species (ROS) due to interrupted photorespiration (Moreno et al., 2005). Therefore, the observed phenotype of *AtSHM1* gene in affecting plant defense abilities likely resulted from abnormal photorespiration.

The evolutionary mechanism of *Rhg4* gene conferring resistance to SCN in soybean, however, has nothing to do with photorespiration. Belonging to the subgroup Ia, *rhg4*-encoded SHMT enzyme likely works in cytosol instead of in mitochondria. Similar to *AtSHM1*, the soybean *rhg4* gene also has a closely related homolog, *rhg4h*, with only the *rhg4* gene involved in SCN resistance. It drives us to think that plants may rely on repeated duplications of *SHMT* genes to enhance their regulatory abilities on one-carbon metabolism, and in some cases, one of the duplicated *SHMT* gene became a defense-related gene. Interestingly, all other eight dicot species investigated by this study retained two copies of sublineage Ia *SHMTs* in their genomes, and their potential roles, e.g. in defense, have not been explored.

The functional differentiation of SHMTs among the different sublineages has also not been well investigated, although available evidence showed that compared to the cytosolic isoform of AtSHM4, the mitochondrial isoforms (AtSHM1 and AtSHM2) exhibited higher enzyme activities in the presence of monoglutamylated folate substrates (Wei et al., 2013). It could be assumed that after ~475 million years evolution in land plants, the four early diverged sublineages of SHMTs had adapted to their own cellular compartmental environments, e.g., to deal with different folate substrates, by fixing certain non-synonymous substitutions. In this study, when examining the position 358 critical for SCN resistance, we found that all sublineage Ia SHMTs have an Asn residue, while other sublineages possess Lys (Ib), Ala (IIa), and Ser (IIb), respectively. For another residue at position 130, the Pro is conservatively maintained in all investigated SHMTs. These data suggested to us that in nature, these two residues are probably functionally important for SHMT enzymes and plants would not afford to mutate them easily. The residue changes (P130R and N358Y) that are critical for the establishment of SCN resistance in certain soybean cultivars, therefore, may just represent an occasional event.

To finally elucidate when those two residue changes occurred in soybean evolution, we successfully amplified both *rhg4* and *rhg4h* genes from 33 cultivated and 68 wild soybeans. On one hand, these two duplicated gene homologs did show evidence of undergoing different functional constraints. Among the eight substitution sites detected in *rhg4* gene, six resulted in amino acid changes, showing a rate significantly deviated from that between *rhg4* and *rhg4h* genes; while the *rhg4h* gene data (one non-synonymous out of six substitution sites) showed no significant deviation. On the other hand, among a total of 20 soybean *rhg4* gene haplotypes detected by this study, the SCN-resistant allele, *Rhg4*, represented by haplotype 4, was only observed in a few cultivars, such as in *Kefeng No. 1* (C01), *Peiking* (C13). A highly similar allele, represented by haplotype 3, is identical to *Rhg4* at all other positions except the residue 130 and 358 (**Figure 3**). This *Rhg4*-similar allele was observed in wild soybeans collected from both south and north china regions at a frequency of 7.4%. Moreover, this allele was also detected in a cultivar, PI88788. As previously mentioned, the residues on position 130 and 358 were highly conservative among different plants, illustrating that they are reluctant to mutate in nature conditions. The presence of haplotype 3, but absence of haplotype 4, in the 68 investigated wild soybeans seems consistent with such conservation pattern. Taken together, the data obtained by this study supported that the SCN-resistant allele, *Rhg4* (haplotype 4), accumulated two more critical substitutions (C to G at nucleotide 389 and A to T at nucleotide 1165) on the foundation of the haplotype 3 allele, a process likely occurred during soybean domestication or improvement process.

In summary, the present study performed sequence analysis of 117 *SHMT* genes of 18 representative plants and reconstructed its phylogeny. Two groups and four subgroups of the *SHMT* gene family were revealed. In subgroup Ia, two soybean genes were detected, which were derived from a whole genome duplication event in the *Glycine* lineage. The two duplicated *rhg4* and *rhg4h* genes underwent differential degrees of functional constraint in the soybean populations, with the *rhg4* gene accumulating more non-synonymous substitutions. The SCN-resistant allele, *Rhg4*, was not detected in wild soybeans; however, a closely related allele showed a frequency of 7.4%, supporting that SCN resistance recently emerged in soybean via artificial selection.

## Author Contributions

BW, X-YW, L-WL, AC, and Y-JZ designed and performed the phylogenetic analysis. X-YW, G-CZ, Y-XC, PW, F-FM, and MW collected soybean samples. X-YW, G-CZ, Y-XC, and C-CL performed PCR analysis. BW, Y-YH, X-YW, and G-CZ performed data analysis. BW and X-YW wrote the manuscript. J-QC and BW conceived and directed the project.

## Conflict of Interest Statement

The authors declare that the research was conducted in the absence of any commercial or financial relationships that could be construed as a potential conflict of interest.

## Footnotes

1 http://phytozome.jgi.doe.gov/pz/portal.html

2 http://congenie.org/
