# Supplementary material for: Soybean Cyst Nematode Resistance Emerged via Artificial Selection of Duplicated Serine Hydroxymethyltransferase Genes
Source: Front Plant Sci. 2016 Jul 8;7:998. doi: 10.3389/fpls.2016.00998 (PMC4937839; doi:10.3389/fpls.2016.00998)
Supplement: Supplementary file 1 [file Image_1.PDF]

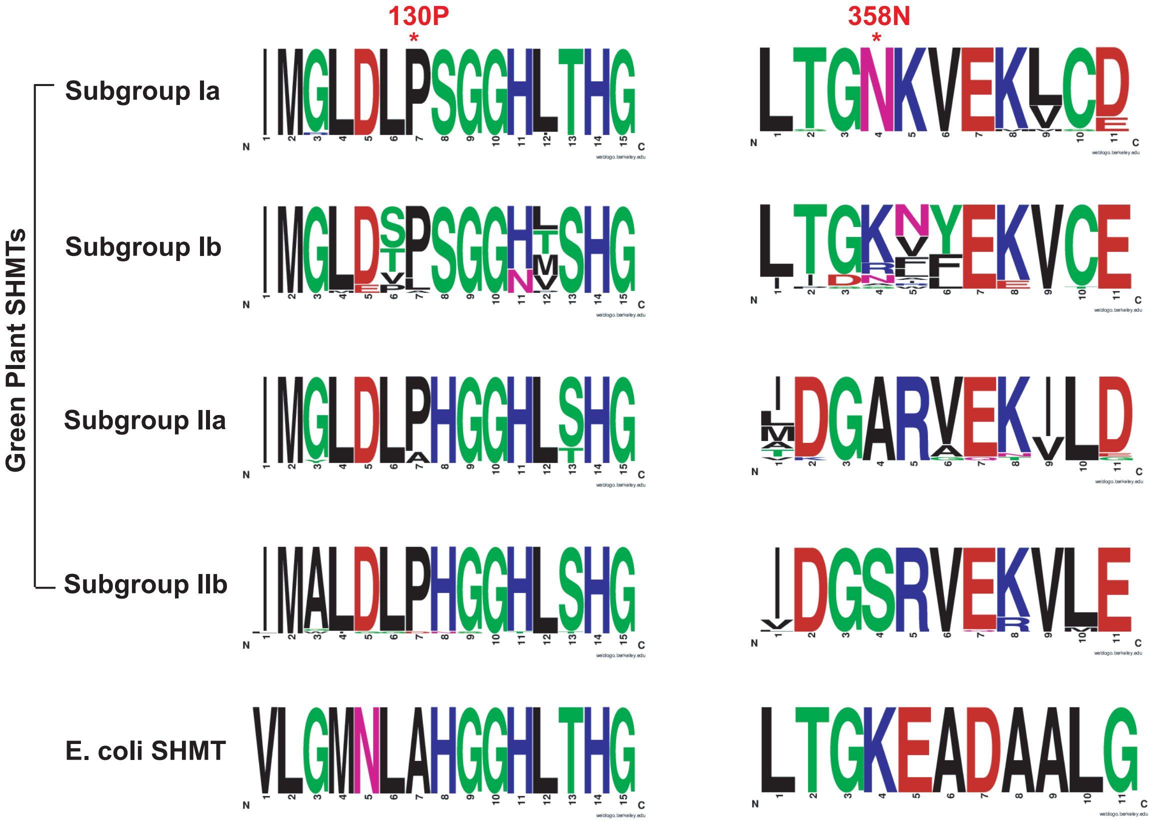

Figure S1. Conservation of amino acid sequences around positions 130 and 358 among the four subgroups of plant SHMTs.
