# Supplementary material for: Soybean Cyst Nematode Resistance Emerged via Artificial Selection of Duplicated Serine Hydroxymethyltransferase Genes
Source: Front Plant Sci. 2016 Jul 8;7:998. doi: 10.3389/fpls.2016.00998 (PMC4937839; doi:10.3389/fpls.2016.00998)
Supplement: Supplementary file 2 [file Table_1.DOC]

**Table S1. SHMT genes detected in 18 representative plant genomes.**

| Organism | Version | Number | Gene | Phylogenetic  group |
| --- | --- | --- | --- | --- |
| *Chlamydomonas reinhardtii* | v5.5 | 3 | Chlre06g293950  Chlre09g411900  Chlre16g664550 | I  IIa  IIb |
| *Ostreococcus lucimarinus* | v2.0 | 3 | Ostlu_30421  Ostlu_37794  Ostlu_29554 | I  IIa  IIb |
| *Physcomitrella patens* | v3.1 | 9 | Phypa_3c9_13200  Phypa_3c15_6270  Phypa_3c4_11810  Phypa_3c4_20570  Phypa_3c12_14950  Phypa_3c13_1080  Phypa_3c13_1100  Phypa_3c1_27990  Phypa_3c14_9630 | Ia  Ia  Ib  Ib  Ib  IIa  IIa  IIb  IIb |
| *Selaginella moellendorffii* | v1.0 | 4 | Selmo_166496  Selmo_90314  Selmo_232016  Selmo_270566 | Ia  Ib  IIa  IIb |
| *Picea abies* | v1.0 | 5 | Picab_11357  Picab_10428694  Picab_169071  Picab_730617  Picab_99276 | Ia  Ib  IIa  IIa  IIb |
| *Amborella trichopoda* | v1.0 | 4 | Ambtr_00013.279  Ambtr_00070.125  Ambtr_00050.66+67  Ambtr_00046.151 | Ia  Ib  IIa  IIb |
| *Oryza sativa* | v7.0 | 5 | Orysa11g26860  Orysa12g22030  Orysa01g65410  Orysa05g35440  Orysa03g52840 (OsSHM1) | Ia  Ia  Ib  Ib  IIb |
| *Brachypodium distachyon* | v2.1 | 4 | Bradi4g08097  Bradi4g19470  Bradi2g56557  Bradi1g09300 | Ia  Ia  Ib  IIb |
| *Sorghum bicolor* | v2.1 | 4 | Sorbi005G113300  Sorbi008G144800  Sorbi003G373600  Sorbi001G097100 | Ia  Ia  Ib  IIb |
| *Arabidopsis thaliana* | TAIR10 | 7 | Arath4G13890 (AtSHM5)  Arath4G13930 (AtSHM4)  Arath1G22020 (AtSHM6)  Arath1G36370 (AtSHM7)  Arath4G32520 (AtSHM3)  Arath4G37930 (AtSHM1)  Arath5G26780 (AtSHM2) | Ia  Ia  Ib  Ib  IIa  IIb  IIb |
| *Arabidopsis lyrata* | v1.0 | 7 | Araly_493523  Araly_947030  Araly_472436  Araly_922779  Araly_491471  Araly_489509  Araly_490821 | Ia  Ia  Ib  Ib  IIa  IIb  IIb |
| *Brassica rapa* | v1.3 | 11 | Brara.D00684  Brara.H00475  Brara.H00601  Brara.H02257  Brara.I03353  Brara.A00543  Brara.H01310  Brara.A00108  Brara.G02412  Brara.G03040  Brara.H01745 | Ia  Ia  Ib  Ib  Ib  IIa  IIa  IIb  IIb  IIb  IIb |
| *Medicago truncatula* | Mt4.0v1 | 8 | Medtr3g084310  Medtr8g081510  Medtr2g006570  Medtr3g073470  Medtr2g018290  Medtr7g108420  Medtr5g030950  Medtr5g067060 | Ia  Ia  Ib  Ib  IIa  IIa  IIb  IIb |
| *Glycine max* | Wm82.a2.v1 | 14 | Glyma05G152100(rhg4h)  Glyma08G108900 (rhg4)  Glyma04G254300  Glyma06G107800  Glyma08G187800  Glyma12G170300  Glyma13G222300  Glyma15G089900  Glyma16G108100  Glyma02G217100  Glyma08G274400  Glyma09G202000  Glyma14G184500  Glyma18G150000 | Ia  Ia  Ib  Ib  Ib  Ib  IIa  IIa  IIa  IIb  IIb  IIb  IIb  IIb |
| *Phaseolus vulgaris* | v1.0 | 7 | Phavu001G266600  Phavu003G051100  Phavu006G216000  Phavu009G026400  Phavu006G168300  Phavu003G286600  Phavu008G184200 | Ia  Ia  Ib  Ib  IIa  IIb  IIb |
| *Mimulus guttatus* | v2.0 | 8 | Mimgu.G00772  Mimgu.N00420  Mimgu.B00818  Mimgu.N01048  Mimgu.A00148  Mimgu.H00289  Mimgu.N02834  Mimgu.O00441 | Ia  Ia  Ib  Ib  IIa  IIb  IIb  IIb |
| *Solanum lycopersicum* | iTAGv2.3 | 7 | Solly05g053810  Solly12g098490  Solly01g104000  Solly08g065490  Solly12g095930  Solly02g091560  Solly04g076790 | Ia  Ia  Ib  IIa  IIa  IIb  IIb |
| *Solanum tuberosum* | v3.4 | 7 | Soltu_400011868  Soltu_400040717  Soltu_400061157  Soltu_400005611  Soltu_400075571  Soltu_400016363  Soltu_400078666 | Ia  Ia  Ib  IIa  IIa  IIb  IIb |
