# Supplementary material for: Soybean Cyst Nematode Resistance Emerged via Artificial Selection of Duplicated Serine Hydroxymethyltransferase Genes
Source: Front Plant Sci. 2016 Jul 8;7:998. doi: 10.3389/fpls.2016.00998 (PMC4937839; doi:10.3389/fpls.2016.00998)
Supplement: Supplementary file 3 [file Table_2.DOCX]

**Table S2. Soybean materials used in this study, including 33 cultivars and 68 wild soybeans.**

| **Soybean No.** | **Names** | **Sources** |
| --- | --- | --- |
| C01 | Kefeng No. 1 | China |
| C02 | Qihuang No. 1 | China |
| C03 | Davis | USA |
| C04 | Kwanggyo_1 | Korea |
| C05 | Zaoshu18 | China |
| C06 | 8101 | China |
| C07 | Youbian30 | China |
| C08 | Nannong1138-2 | China |
| C09 | Xudou No. 1 | China |
| C10 | CNS_1 | USA |
| C11 | CNS _2 | USA |
| C12 | Jindou No. 1 | China |
| C13 | Peking | China |
| C14 | Juxuan 23 | China |
| C15 | Columbia_1 | USA |
| C16 | Xudou No. 2 | China |
| C17 | Jiunong 21 | China |
| C20 | York (PI553038) | USA |
| C21 | Marshall（PI548693) | USA |
| C22 | Kwanggyo_2 | USA |
| C23 | Ogden (PI548477) | USA |
| C24 | Raiden (PI360844) | USA |
| C25 | Suweon97 (PI483084) | USA |
| C26 | Tousan50 (PI507389) | USA |
| C27 | L29 | USA |
| C28 | V94-5152 | USA |
| C30 | Essex | USA |
| C37 | Harosoy (PI548573) | Japan |
| C40 | Hourei (PI561394) | Japan |
| C41 | J05 | China |
| C42 | Columbia_2 (PI548317) | USA |
| C43 | PI88788 | China |
| C44 | TieFeng25 (ZDD07695) | China |
| W46 | NE001 | Northeast, China |
| W49 | NE002 | Northeast, China |
| W51 | AHSJHM001 | South Jiuhua Mt., Anhui, China |
| W52 | AHSJHM002 | South Jiuhua Mt., Anhui, China |
| W53 | AHSNG001 | South Ningguo, Anhui, China |
| W54 | FJFZ001 | Fuzhou, Fujian, China |
| W55 | FJJY001 | Jianyang, Fujian, China |
| W56 | FJLC001 | Liancheng, Fujian, China |
| W57 | FJLC002 | Liancheng, Fujian, China |
| W58 | FJTN001 | Taining, Fujian, China |
| W59 | FJXP001 | Xiapu, Fujian, China |
| W60 | GX001 | Guangxi, China |
| W62 | GZRJ001 | Rongjiang, Guizhou, China |
| W63 | HBCY001 | Chongyang, Hubei, China |
| W64 | HBHF001 | Hefeng, Hubei, China |
| W65 | HBLC001 | Lichuan, Hubei, China |
| W66 | HBYE001 | Yien, Hubei, China |
| W67 | HNLY001 | Liuyang, Hunan, China |
| W68 | JSJJ001 | Jingjiang, Jiangsu, China |
| W69 | JXJJ001 | Jiujiang, Jiangxi, China |
| W70 | JXXS001 | Xiushui, Jiangxi, China |
| W71 | JXYJ001 | Yujiang, Jiangxi, China |
| W72 | JXZX001 | Zixi, Jiangxi, China |
| W73 | SC001 | Sichuan, China |
| W74 | SCLP001 | Liangping, Sichuan, China |
| W75 | YN001 | Yunnan, China |
| W76 | ZJLS001 | Lishui, Zhejiang, China |
| W77 | ZJLS002 | Lishui, Zhejiang, China |
| W78 | ZJLA001 | Linan, Zhejiang, China |
| W79 | ZJSX001 | Shengxian, Zhejiang, China |
| W80 | ZJTT001 | Tiantai, Zhejiang, China |
| W81 | HLJXK001 | Xunke, Heilongjiang, China |
| W82 | HLJHG001 | Hegang, Heilongjiang, China |
| W83 | HLJDLH001 | Dalianhe, Heilongjiang, China |
| W85 | HLJSL001 | Shuileng, Heilongjiang, China |
| W86 | HLJCL001 | Cuiluan, Heilongjiang, China |
| W87 | HLJLK001 | Linkou, Heilongjiang, China |
| W88 | HLJSW001 | Sunwu, Heilongjiang, China |
| W89 | HLJEC001 | Echeng, Heilongjiang, China |
| W90 | HLJFJ001 | Fujin, Heilongjiang, China |
| W92 | HLJYC001 | Yichun, Heilongjiang, China |
| W93 | HLJSHBL001 | Shuihua, Heilongjiang, China |
| W94 | HLJJX001 | Jixi, Heilongjiang, China |
| W95 | HLJHLQF001 | Hulin, Heilongjiang, China |
| W97 | HLJHL001 | Hailun, Heilongjiang, China |
| W98 | HLJMEM001 | Maoer Mt., Heilongjiang, China |
| W99 | HLJMS001 | Mingshui, Heilongjiang, China |
| W100 | HLJTJQDL001 | Tongjiang, Heilongjiang, China |
| W102 | HLJSGL001 | Shangganling, Heilongjiang, China |
| W103 | HLJNHTH001 | Nahetaihe, Heilongjiang, China |
| W104 | HLJYCFL001 | Fenglin, Yichun, Heilongjiang, China |
| W105 | HLJYY001 | Youyi, Heilongjiang, China |
| W106 | HLJKD001 | Kedong, Heilongjiang, China |
| W107 | HLJTH001 | Tahe, Heilongjiang, China |
| W108 | HLJHM001 | Huma, Heilongjiang, China |
| W109 | HLJSB001 | Shuibing, Heilongjiang, China |
| W120 | JSYZGC001 | Guochun, Yangzhou, Jiangsu, China |
| W121 | JSTZQH001 | Qinhu, Taizhou, Jiangsu, China |
| W122 | JSTZQH002 | Qinhu, Taizhou, Jiangsu, China |
| W123 | JSYCDT001 | Dongtai, Yancheng, Jiangsu, China |
| W129 | HLJHEB001 | Haerbing, Heilongjiang, China |
| W130 | HLJHEB002 | Haerbing, Heilongjiang, China |
| W131 | HLJWDLC001 | Wudalianchi, Heilongjiang, China |
| W132 | HLJWDLC002 | Wudalianchi, Heilongjiang, China |
| W133 | HLJMDJJPH001 | Jingpohu, Mudanjiang, Heilongjiang, China |
| W134 | HLJBAHLTX001 | Tongxing, Hailun, Heilongjiang, China |
| W135 | HLJHLHB001 | Heibei, Hailun, Heilongjiang, China |
| W136 | HLJHLDS001 | Dongsheng, Hailun, Heilongjiang, China |
